# The effect of psychological interventions targeting overweight and obesity in school-aged children: a systematic review and meta-analysis

**DOI:** 10.1186/s12889-023-16339-7

**Published:** 2023-08-03

**Authors:** Fereshteh Baygi, Shirin Djalalinia, Mostafa Qorbani, Anders Larrabee Sonderlund, Merethe Kirstine Kousgaard Andersen, Trine Thilsing, Berit Lilienthal Heitmann, Jesper Bo Nielsen

**Affiliations:** 1 Research Unit of General Practice, Department of Public Health, University of Southern Denmark, Odense, Denmark; 2 Ministry of Health and Medical Education, Deputy of Research & Technology, Tehran, Iran; 3 Non-Communicable Diseases Research Center, Alborz University of Medical Sciences, Karaj, Iran; 4 Research Unit for Dietary Studies, the Parker Institute, Frederiksberg and Bispebjerg Hospital, Frederiksberg, Denmark; 5 Section for General Practice, Department of Public Health, University of Copenhagen, Copenhagen, Denmark

**Keywords:** School-aged Children, Meta-analysis, Psychological Interventions, Overweight, Obesity

## Abstract

**Background:**

Multi-component psychological interventions may mitigate overweight and obesity in children and adolescents. Evidence is, however, scattered on the effectiveness of such interventions. This study aims to review the available evidence on the effectiveness of multi-component psychological interventions on anthropometric measures of school-aged children with overweight or obesity.

**Methods:**

We systematically searched international databases/search engines including PubMed and NLM Gateway (for MEDLINE), Web of Science, SCOPUS, and Google Scholar up to November 2022 for relevant articles pertaining to psychological weight-loss interventions targeting school-aged children. Two reviewers screened and extracted pertinent data. The quality of included studies was assessed using the Cochrane Risk of Bias Tool for Randomized Trials. Random effect meta-analysis was used to calculate, and pool standardized mean differences (SMD). We distinguished between intervention and maintenance effects. Intervention effects were defined as the mean change in outcome measurement detected between baseline and post-treatment. Maintenance effects were defined as the mean change in outcome measurement between post-treatment and last follow-up.

**Results:**

Of 3,196 studies initially identified, 54 and 30 studies were included in the qualitative and quantitative syntheses, respectively. Most studies reported on group-based interventions. The significant effects of intervention on BMI z-score (SMD -0.66, 95% CI: -1.15, -0.17) and WC (SMD -0.53, 95% CI: -1.03, -0.04) were observed for interventions that centered on motivational interviewing and cognitive behavioral therapy, respectively. Mean BMI and WC did not differ significantly between post-treatment and last follow-up measurement (maintenance effect), indicating that an initial weight loss obtained through the intervention period could be maintained over time.

**Conclusions:**

Findings indicate that motivational interviewing and cognitive behavioral therapy as interventions to reduce BMI z-score (generalized obesity) and waist circumference (abdominal obesity) are effective and durable. However, detailed analyses on individual components of the interventions are recommended in future effectiveness studies.

**Supplementary Information:**

The online version contains supplementary material available at 10.1186/s12889-023-16339-7.

## Background

The global burden of non-communicable diseases (NCDs) has increased over recent years [1, 2]. NCDs accounted for 7.1 million additional deaths in 2019 compared to 2009 globally [3]. Obesity is a prominent risk factor for many NCDs, including cardiovascular diseases, type 2 diabetes, cancer, disability, and death [1–3]. Over two thirds of the key drivers of NCDs (e.g., unhealthy diet, physical inactivity, obesity) are formed or emerge during childhood and adolescence [4]. Therefore, prevention must start with this age group at both national and global levels.

Recent estimates based on 2416 data sources showed that between 1975 and 2016, the global prevalence of obesity increased from 0.7 to 5.6% in girls and from 0.9 to 7.8% among boys aged 5–19 years [5]. There is also evidence showing a positive association between childhood obesity and the development of NCD risk factors in adulthood [2]. This underscores the urgent need to address overweight and obesity already in childhood.

Several studies indicate that diet therapy, exercise, and education, along with parent education, may be effective approaches to reduce obesity among children and adolescents [6–9]. There is also evidence suggesting that combining behavioral (e.g., diet or physical activity interventions) and psychological methods (e.g., cognitive behavioral therapy or motivational interviewing) in multi-component interventions might enhance weight reduction among children with obesity above and beyond single-component interventions [10, 11]. In addition, this evidence indicates that maintenance of weight loss over time might be more difficult to achieve than the initial weight reduction. So, the main goal of psychological interventions in obesity management is to provide effective interventions to make changes that are durable [10, 11]. However, existing evidence on the efficacy of these types of multi-component interventions in terms of weight-loss maintenance is somewhat scattered—especially related to child populations [10]. To our knowledge there are currently no published systematic reviews or meta-analyses that have addressed effectiveness of psychological intervention on obesity management. Therefore, this study aims to review the existing evidence on the efficacy of child weight-loss interventions that include a psychological intervention component.

The present study addresses the following research questions: 1) What kind of psychological interventions have been applied so far targeting overweight and obesity in school-aged children? 2) What are the most effective psychological intervention methods for reducing overweight and obesity among school-aged children? 3) What are the strengths and limitations of each of the intervention designs and/or methods in terms of efficacy, durability, and implementation?

### Definition of psychological interventions

In the broadest terms, psychological interventions relate to non-pharmacological interventions that target cognitive, behavioral, emotional, interpersonal, social, or environmental factors to improve a particular health outcome or condition [12]. They involve psychological therapies (e.g., cognitive behavioral therapy), education and social support approaches, social environment and norm-based strategies, or a combination of these [12].

Psychological therapy in overweight and obesity may include, for example, cognitive behavioral therapy (CBT), motivational interviewing (MI), or acceptance therapy [13]. Psychological interventions may be individual, or group based [12]. For maximum effect, these interventions usually are applied in the context of a multi-component weight loss program (e.g., dietary and exercise strategies as a behavioural therapy) [13].

## Methods

### Identification of relevant studies

**Fig. 1:**
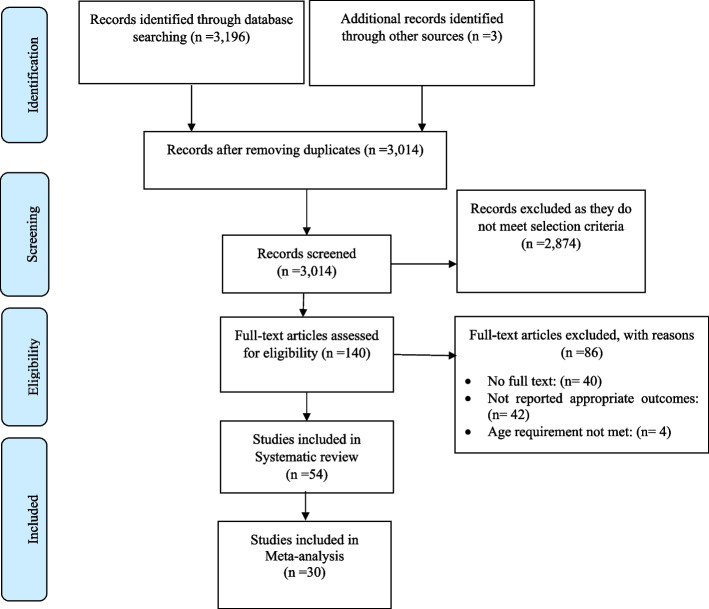
Flow diagram of study selection

We conducted the present study according to the Preferred Reporting Items for Systematic Reviews and Meta-Analysis guideline (PRISMA) (Fig. 1) [14]. The study protocol has been published in the International Prospective Register of Systematic Reviews (PROSPERO), registration number: CRD42022309438.

The search strategy was developed to target three key focus areas: Psychological interventions, overweight/obesity, and school-aged children (See Additional file 1 for the full search strategy). The search included studies published up to and including November 2022. No language or geographical limitation were applied. The optimal sensitivity of searching for articles, was reached by simultaneous search of the most comprehensive databases/ search engines including PubMed and NLM Gateway (for MEDLINE), Web of Science (ISI/WOS), SCOPUS, and Google Scholar. Further, reference lists of the included publications or reviews were studied to identify potentially eligible studies that were missed in the database searches. Relevant book chapters were also reviewed for data. If multiple publications were based on a single study, just one publication was included. Duplicates and non-peer reviewed publications were excluded.

### Primary outcomes

Change in anthropometric measures (weight, body mass index (BMI), BMI z-score, waist circumference (WC), waist-to-height ratio (WhtR), percentage overweight) represented the primary outcome.

### Secondary outcomes

Changes in body composition, blood pressure, biomarkers of metabolic-syndrome, behavioral measures (e.g., dietary intake, physical activity), and measures of quality of life (e.g., perceived physical and mental health) were secondary outcomes.

### Inclusion and exclusion criteria

#### The inclusion criteria are as follow

1- The study population was limited to school-aged children (6–18 years old). 2- Participants in each study had overweight or obesity according to any parameter (e.g., BMI, BMI z-score, WC, WhtR, percent body fat). 3- Studies comprised randomized controlled trials (RCTs) or quasi-randomized controlled trials (QRCTs) which had applied any kind of psychological interventions for weight loss in school-aged children with overweight or obesity.

#### The exclusion criteria are as follow

1- Studies without a primary outcome of obesity reduction 2- Observational studies/ non-randomized controlled trials 3- Studies on participants with prevalent disease, eating disorder (e.g., anorexia nervosa) or special needs besides overweight interventions 4- Studies that had combined pharmacological intervention on obesity with psychological interventions 5- Studies without sufficient information to determine eligibility.

### Quality assessment

Two researchers conducted the systematic literature review, study-quality assessment, and the data extraction. Any inter-rater discrepancies were resolved by a third party. The extracted data included author and year of publication, country, population characteristics (e.g., age), and methodology (e.g., study design, sample size, type of psychological intervention/theory, duration of intervention, and outcomes.

The quality assessment for each of the included studies was done using the Cochrane Risk of Bias tool (RoB_2_) for randomized controlled trials [15]. The RoB_2_ comprises five domains of potential bias. These include bias in the randomization process, bias due to deviations from intended interventions, bias due to missing outcome data, bias in outcome measurement, and bias in the reporting of results [15]. Employing the validated RoB_2_ algorithm (available at https://methods.cochrane.org/bias/), each domain was assessed in terms of three levels of risk of bias: low risk, some concerns, or high risk.

### Data extraction

Outcome data were extracted from baseline, post-treatment, and subsequent follow-up measurement. Intervention effects were defined as the mean difference in outcome measurement detected between baseline and post-treatment in both control and intervention groups. Maintenance (effectiveness) was defined as mean difference in outcome between the post-treatment and last follow up measurement. If multiple follow-up data were provided, the last follow-up data were used for estimation of maintenance effect size.

### Statistical analysis

Heterogeneity among studies was assessed using the chi-square-based Q-test and the I-squared statistic. A random-effects meta-analysis model using the Hedges method [16] was used when heterogeneity was statistically significant (Q-test < 0.1).

Standardized mean differences (SMDs) and 95% confidence intervals (CIs) were used to determine intervention effect sizes in terms of the primary and secondary outcomes.

Subgroup analysis was performed with respect to intervention duration (≤ 6 month, > 6 month), age category (≤ 12-year-old, > 12-year-old), intervention design (group-based, individual-based), intensity of the intervention (low, moderate, high), and type of control group (active, passive, routine care, and no-intervention).

Hours of contact was calculated as a proxy for intensity of the interventions. The figures were categorized as very low (< 10 h), low (10–25 h), moderate (26–75 h), or high (> 75 h) [17].

Control group type was determined based on the study information.

Publication bias was assessed with Egger's test at a significance level of *p* < 0.1.

A meta-regression analysis was conducted to find the sources of heterogeneity. All analyses were conducted using Stata 17 (version 17; Stata Corp, College Station, Texas).

A limited number of studies included secondary outcomes (e.g., quality of life, dietary outcomes). Of these studies, heterogeneity across studies in terms of methodology, outcome measurement, and type of intervention precluded an accurate meta-analysis of these outcomes. For this reason, our findings that relate to secondary outcomes are presented only but qualitatively.

## Results

### Findings from systematic review

#### Study selection process

A total of 3,196 studies was identified in the initial database search. An additional three articles were identified through citation searching. After deduplication, 3,014 articles remained. A total of 2,874 articles was excluded based on screening titles and abstracts. Of the remaining 140 studies, 86 were excluded after the full-text review. The most common reasons for exclusion related to study design (non-RCT), the intervention lacking psychological components and/or not targeting a relevant outcome, or not providing sufficient information about the studied population (Fig. 1). Ultimately, 54 studies were eligible for inclusion in the systematic review [7, 18–70].

#### Study characteristics

The characteristics of the included studies on the efficacy of psychological interventions targeting overweight and obesity in school-aged children are demonstrated in Additional file 2. Most of the studies (41 out of 54 included studies) were published within the recent 10 years (as of 2023), indicating increasing focus on this topic as a global public health challenge.

Most studies (*n* = 31) were conducted in the US [18, 20, 22–24, 28, 30, 32, 34, 36–38, 40, 41, 43, 45–49, 51, 54, 55, 60, 61, 65, 66, 68–70], followed by Iran [19, 29, 57, 62], UK [33, 53, 63], Denmark [31, 35], the Netherlands [42, 58], China [39, 67], Israel [27, 52], Germany [7], Spain [21], Iceland [25], Turkey [26], Norway [44], Belgium [50], Switzerland [64], Australia [56], and Mexico [59].

Sample sizes ranged from 27 participants in a call-based intervention [32] to 549 in a community and clinic-based intervention [39]. Cognitive therapy alone or in combination with other behavioral methods was the most applied approach in the included studies [18–22, 27–30, 34, 36, 37, 42, 44, 48, 50, 55, 56, 58, 61, 64, 65, 68]. BMI z-score and BMI were the most frequently used outcome measures [18, 20, 21, 23–59, 61–70].

All studies applied multi-component psychological interventions based on either nutritional and/or physical activity programs for weight reduction. Quality of life (QL) [18, 19, 47, 54, 60, 63], dietary intakes [18–20, 23, 29, 39, 45, 48, 66], and physical activity (PA) [19–21, 23, 34, 39, 48, 63] were measured using a wide range of assessment tools.

Intervention duration ranged from 1.5 months in a day-camp and home setting intervention [31] to 36 months [20] (intervention setting was not provided by the researchers). Intervention sessions were variable (e.g., daily, or monthly basis). Follow-up time ranged from 2.5 months [31] to 24 months [27, 35]. In more than half of the studies (35/54), intervention groups were compared with control groups, while in the rest of the studies two or three intervention groups were compared. The reported interventions were implemented by people from a wide range of academic and professional backgrounds (e.g., school nurse, psychologist) and educational levels (e.g., master-level instructors, professional intervention delivery agent). Overall, most studies used professionals to implement the interventions, whereas in one study [51], intervention was delivered by using, an automated interactive voice response system (machine).

#### Type of psychological interventions

As illustrated in Additional file 2, different types of psychological interventions were applied and evaluated in the reviewed studies (e.g., cognitive behavioral, motivational interviewing).

#### Psychological intervention designs

**Fig. 2:**
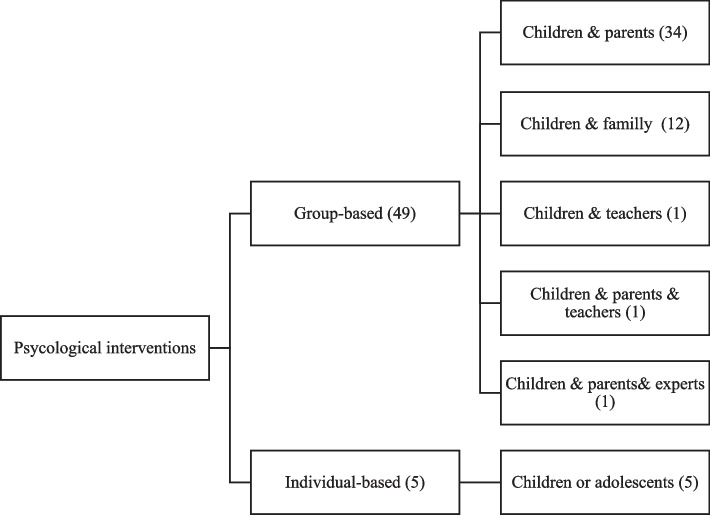
Psychological interventions designs targeting overweight, and obesity in school-aged children (based on studies included in systematic review)

Figure 2 summarizes different psychological intervention designs used in the included studies. Group-based interventions represented the most common intervention design. Most interventions targeted both children and their parents [18–21, 24–27, 30, 32, 35–39, 41, 43, 46, 48, 50, 51, 55, 57–59, 61, 62, 64, 66–70]. Individual designs were applied in five studies that focused on children or adolescents [23, 28, 33, 34, 54].

#### Strengths and limitations of the intervention designs and methods

Table 1 illustrates strengths and limitations of different psychological designs adapted from [21, 58, 71–82] additionally with authors’ opinion. Furthermore, the following paragraph provide an overview on the main strengths and limitations of the most prominent psychological intervention methods- adapted from [83–85], additionally with authors’ opinion.

**Table 1:** Strengths and limitations of psychological intervention designs for treatment of school-aged children with overweight or obesity (Adapted from [21, 58, 71–82] additionally with authors’ opinion)

Methods	Theoretical grounding	Limitations	Strengths
Group-based [21, 58, 71–79]	Psychosocial; cognitive behavioral; social cognitive theory; mindfulness	• Efficacy depends on entire family unit/ group of people • Requires longer timeframes and extensive training of clinical staff • Requires rigid structure/guidelines • Difficult to tease out effectiveness of individual intervention components • Works best in conjunction with other approaches	• May be more effective than cognitive behavioral treatment that does not include family • Appropriate for young and older children • Group-based format facilitates social support and shared social identity (both correlate with health) • Focus often on social environment as well as individual behavior & motivation
Community-based [80, 81]	Psychosocial; cognitive behavioral; motivation theory	• Difficult to implement (requires community involvement, primary care, schools, training of staff, parents, etc.) • May not be as effective in younger populations (pre-treen) • Interventions may take large-group approach (classroom) which may not reach everyone • Requires longer timeframe to implement and evaluate • Requires regular evaluation • Most effective with structural change (e.g., norm-based plus restrictions on junk food in school)	• Prevention and treatment typically in focus • Targeting both norm and structural change • May be more useful in underserved communities • Norm-based interventions have proven efficacious in multiple other health- and non-health-related settings • Effective for children with autonomy as well as parents/care givers/teachers of younger, more dependent children • Focus predominantly on social/structural environment as determinant of individual behavior & motivation • Often holistic approach to health
Individual-based [21, 78, 82]	Psychosocial; cognitive behavioral; dual-process cognition; motivational interviewing	• May be less efficacious in younger and less autonomous populations • Difficult to tease out effectiveness of individual intervention components • Generally mixed evidence for efficacy • Works best in conjunction with other approaches • Lack of focus on social environment, onus is on individual to change • More expensive than group-based interventions	• Dependent only on individual rather than group/family • Tailored to the individual • More intensive and focused than group- or community-based interventions

As the most frequent method in the reviewed studies, CBT covers a wide range of psychological aspects and social consequences of obesity from anxiety and loneliness to weight loss. It can also facilitate long-term weight-maintenance skills and improve a person’s self-esteem and -image. However, it also has limitation meaning that CBT alone will not work for everyone with obesity. So, it might be combined with other interventions like lifestyle changes to provide better results on obesity management.

MI alone, or in combination with CBT, also featured in the reviewed studies. MI has a strong theoretical foundation that emphasizes the importance for the individual to recognize and internalize why change is necessary and how change can be achieved. However, this highly individualized approach is also a limitation as implementation requires extensive tailoring to the target individual or group.

### Findings from meta-analysis

Of the 54 included studies, 30 were eligible for inclusion in the meta-analysis [7, 18–20, 23, 24, 27–30, 33, 34, 36, 37, 39, 40, 44, 48, 50–54, 57–61, 65, 67]. Reasons for excluding 24 studies from further analysis were due to several issues (e.g., having unclear, insufficient, or missing data). The combined sample size of RCT studies included in the meta-analysis totaled 4093 school-aged children with overweight or obesity.

The pooled intervention effect is shown in Table 2. The intervention effect was statistically significant on all BMI measures (BMI and BMI z-score) (SMD: -0.59, 95% CI: -0.89, -0.20, I^2^ = 96.64), BMI z-score (SMD: -0.39, 95% CI: -0.69, -0.09, I^2^ = 88.60) and BMI (SMD: -0.79, 95% CI: -1.48, -0.09, I^2^ = 97.80). We also detected statistically significant intervention effects on fat mass (SMD: -0.96, 95% CI: -1.54, -0.38, I^2^ = 85.00), body fat (SMD: -0.81, 95% CI: -1.30, -0.33, I^2^ = 69.29), and diastolic blood pressure (SMD: -0.49, 95% CI: -0.83, -0.15, I^2^ = 41.80).

**Table 2:** Intervention effect on outcomes of overweight and obesity among school-aged children in the meta-analysis

Outcome		No. studies	Sample size	Effect size		Heterogeneity assessment		
				**SMD (95% CI)**	***P*-value**	**I-squared %**	**Q-test**	***P*-value**
BMI (kg/m^2^)	z-score	15	2,210	-0.39 (-0.69, -0.09)	0.01	88.6	119.40	< 0.001
	Score	16	2,064	-0.79 (-1.48, -0.09)	0.03	97.8	530.80	0.03
	All measures (Both BMI measures combined)	26	3,417	-0.59 (-0.89, -0.20)	< 0.001	96.64	652.2	< 0.001
WC (cm)		9	1,079	-0.78 (-2.01, 0.45)	0.21	98.7	383.00	< 0.001
WHR		3	375	-.04 (-0.65, 0.58)	0.91	88.8	16.90	< 0.001
Fat mass (kg)		5	418	-0.96 (-1.54, -0.38)	< 0.001	85.0	25.20	< 0.001
Body fat (kg)		3	351	-0.81 (-1.30, -0.33)	< 0.001	69.29	4.66	0.10
Percentage overweight		3	405	1.61 (-4.86, 1.65)	0.33	99.20	226.54	< 0.001
SBP (mm Hg)		2	134	-0.60 (-1.32, 0.12)	0.10	77.03	4.35	0.04
DBP (mm Hg)		2	134	-0.49 (-0.83, -0.15)	0.03	41.8	1.72	0.19

*SMD* Standardized mean difference, *CI* Confidence Interval, *BMI* Body Mass Index, *WC* Waist Circumference, *WHR* Waist to Hip Ratio, *SBP* Systolic Blood Pressure, *DBP* Diastolic Blood Pressure

The pooled maintenance effect of intervention is shown in Table 3. Mean BMI and WC did not differ significantly between post-treatment and last follow-up measurement, indicating that initially lost weight is not regained during a subsequent follow-up period (maintenance effect).

**Table 3:** Maintenance effect of the intervention on outcomes of overweight and obesity among school-aged children in the meta-analysis

**Outcome**		**No. studies**	**Sample size**	**Effect size** ^a^		**Heterogeneity assessment**		
				SMD (95% CI)	*P*-value	I-squared %	Q-test	*P*-value
BMI (kg/m^2^)	BMI z-score	5	611	-0.05 (-0.23, 0.12)	0.57	0.00	2.64	0.62
	BMI	5	941	-0.23 (-0.56, 0.09)	0.16	79.30	22.41	< 0.001
	All measures	9	1,476	-0.16 (-0.33, 0.02)	0.08	57.98	28.03	< 0.001
WC (cm)		4	800	-0.05 (-2.67, 0.57)	0.20	98.75	177.70	< 0.001

*SMD* Standardized mean difference, *CI* Confidence Interval, *BMI* Body Mass Index, *WC* Waist Circumference

^a^ A negative number means that the BMI is reduced after the intervention period

Table 4 shows the results of sub-groups meta-analyses by type, duration, design, and intensity of psychological intervention, population age, and type of control group. In sub-group analyses the significant effect of psychological interventions on BMI was observed in studies conducted on children > 12 years old (SMD = -0.79, 95% CI: -1.57, -0.01), group-based interventions (SMD = -0.98, 95% CI: -1.80, -0.16), intervention of moderate intensity (SMD = -1.40, 95% CI: -2.56, -0.23), and interventions with a passive control group (SMD = -0.41, 95% CI: -0.79, -0.02). Moreover, the significant effect of psychological interventions on BMI z-score was observed in studies with very low (SMD = -0.88, 95% CI: -1.20, -0.56) or moderate intervention intensity (SMD = -0.96, 95% CI: -1.83, -0.10), or studies conducted with treatment-as-usual control group (SMD = -0.86, 95% CI: -1.04, -0.67). Significant effects on WC were observed in studies with low intervention intensity (SMD = -0.34, 95% CI: -0.59, -0.09) or studies conducted with passive control groups (SMD = -0.24, 95% CI: -0.44, -0.04).

**Table 4:** Stratified random effect meta-analysis of effect of intervention on outcomes in target population according to type of psychological intervention, intervention duration, age, intervention design, intensity of intervention, type of control group

	BMI		
	**BMI z-score** SMD (95% CI)	**BMI means (kg/m**^**2**^**)** SMD (95%CI)	**WC (cm)** SMD (95%CI)
**Sub- groups**			
**Type of psychological intervention**			
CBT	-0.31(-0.79, 0.17)	-0.39 (-0.88, 0.09)	-0.53 (-1.03, -0.04)^a^
CBT& MI	0.12 (-0.17, 0.42)	0.12 (-0.17, 0.42)	--------
MI	-0.66 (-1.15, -0.17)^a^	-1.43 (-3.37, 0.49)	-2.12 (-5.09, 0.84)
**Intervention duration**			
≤ 6 month	-0.46 (-0.81, -0.11)^a^	-0.75 (-1.58, 0.06)	-1.27 (-2.58, 0.02)
> 6 month	-0.13 (-0.75, -0.08)^a^	-0.88 (-2.36, 0.59)	0.96 (-0.88, 2.80)
**Age**			
≤ 12-year-old	-0.33 (-0.74, 0.07)	-0.44 (-1.73, 0.84)	-1.85 (-5.09, 1.39)
> 12-year-old	-0.16 (-0.52, 0.20)	-0.79 (-1.57, -0.01)^a^	0.17 (-0.99, 1.34)
**Intervention design**			
Group-based	-0.45 (-0.79, -0.11)^a^	-0.98 (-1.80, -0.16)^a^	-1.11 (-2.29, 0.06)
Individual-based	-0.02 (-0.29, 0.25)	-0.21 (-1.51, 1.09)	-
**Intensity of intervention**			
Very low (< 10 h)	-0.88 (-1.20, -0.56)^a^	-	-
Low (10–25 h)	-0.25 (-1.09, 0.58)	-0.25 (-0.50, 0.00)	-0.34 (-0.59, -0.09)^a^
Moderate (26–75 h)	-0.96 (-1.83, -0.10)^a^	-1.40 (-2.56, -0.23)^a^	-1.86 (-4.07, 0.35)
High (> 75 h)	-	-	0.96 (-0.88, 2.80)
**Type of control group**			
Passive	-0.27 (-0.86, 0.32)	-0.41 (-0.79, -0.02)^a^	-0.24 (-0.44, -0.04)^a^
Active	-0.07 (-0.45, 0.32)	-0.53 (-1.40, 0.33)	0.39 (-1.17, 1.95)
Routine care	-0.86 (-1.04, -0.67)^a^	-1.00 (-3.51, 1.50)	-2.97 (-7.23, 1.29)
No Intervention	-0.62 (-1.72, 0.48)	-1.93 (-2.76, -1.10)^a^	-

*SMD* Standardized mean difference, *CI* Confidence Interval, *BMI* Body Mass Index, *WC* Waist Circumference, *CBT* Cognitive Behavior Therapy, *MI* Motivational Interviewing

^a^Statistically significant

MI and CBT—as effective psychological interventions—significantly reduced BMI z-score (generalized obesity) (SMD = -0.66, 95% CI: -1.15, -0.17) and WC (abdominal obesity) (SMD -0.53, 95% CI: -1.03, -0.04), respectively.

The highest pooled SMD in BMI z-score (SMD -0.96, 95% CI: -1.83, -0.10) was observed in interventions of moderate intensity.

**Fig. 3:**
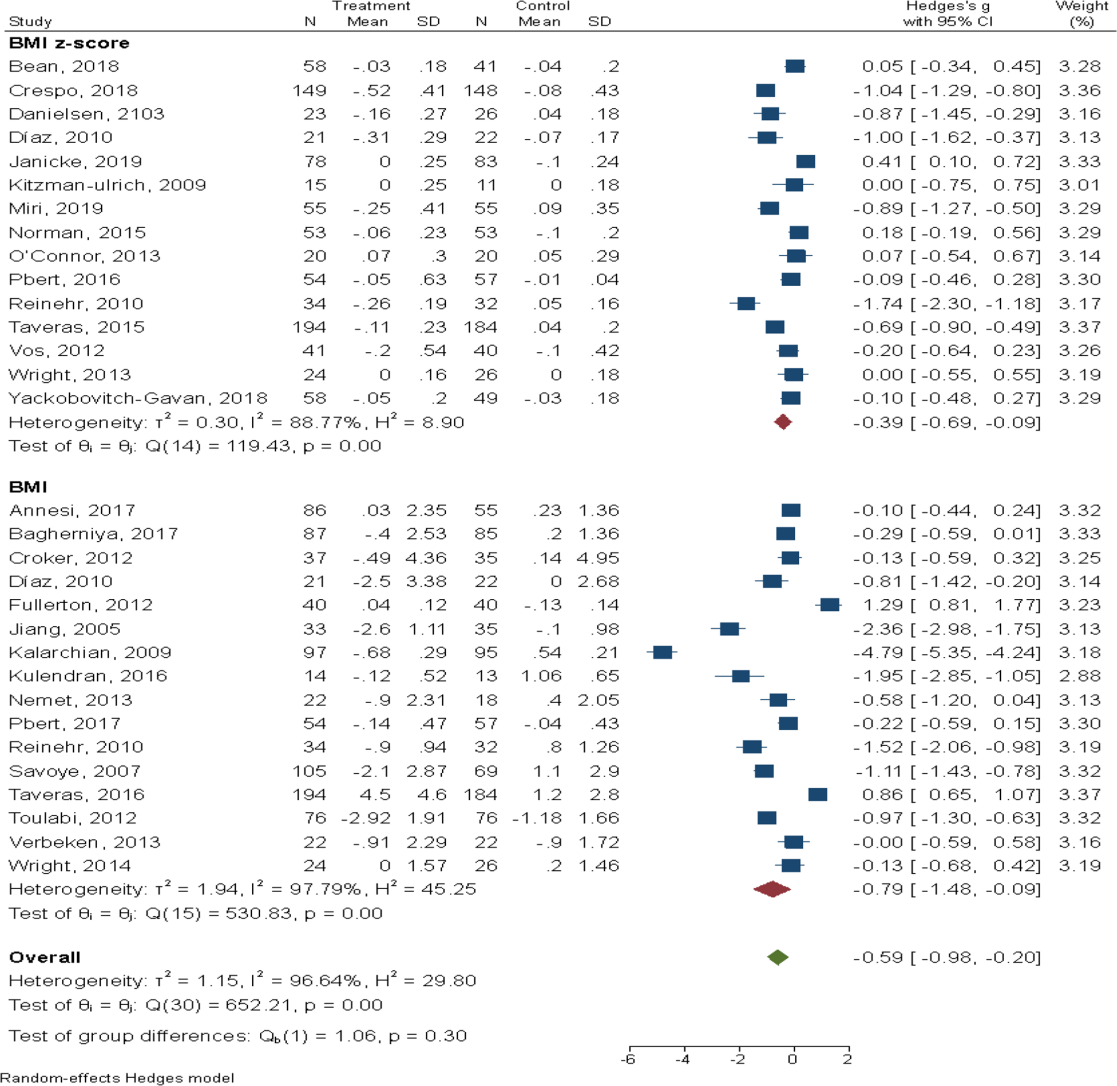
Forest plot of psychological intervention effect on BMI measures in school-aged children with overweight and obesity

Figure 3 shows forest plot of intervention effects on BMI measures in our target population.

### Meta-regression

Sufficient data were available to perform a meta-regression analysis of intervention effect on BMI z-score, BMI, and WC by intervention type, duration, design, and intensity, age, and type of control group. Meta-regression of BMI-z score showed that age (coefficient: -0.77, SE: 0.22; *p* < 0.100), intervention intensity (coefficient: 0.18, SE: 0.08; *p* < 0.100), and type of control group (coefficient: -0.42, SE: 0.14; *p* < 0.100) contributed to the heterogeneity. No sources of heterogeneity were found for BMI and WC.

### Quality of the studies

The kappa statistic for agreement of quality assessment was 0.90. The quality assessment of the included studies is presented in Additional file 2. Of 54 included studies, 22 (41%) were rated as low risk of bias. While eight studies were rated as high risk of bias. For the rest of the studies there were some concerns about the risk of bias.

### Publication bias

The result of Egger’s test revealed no substantial risk of publication bias neither for the intervention effect outcomes, nor for the maintenance ones (*p*-value > 0.1).

## Discussion

Our study revealed that several different types of psychological interventions targeting school-aged children with overweight, or obesity have been trialed. CBT alone or in combination with other behavioral methods was the most frequently applied approach in the reviewed studies.

Selection of an appropriate psychological approach for successful weight management plan depends on creating a good connection with educational interventions [86]. So, researchers, professional health care providers, and general practitioners (GPs) who are working with overweight or obesity of school-aged children are encouraged to consider such conjunctions for an effective weight management plan.

Our findings also revealed that group-based interventions which rely on parents to implement lifestyle strategies were the most common method for managing overweight and obesity in the reviewed studies. This may depend on that parents’ engagement can support and facilitate the process of obesity treatment that requires a combination of strategies for lifestyle and behavioral changes [87]. We also believe that parents are very important for success in any kind of the interventions for weight management by helping children to commit to new healthy lifestyle activities. On the other hand, parents have some level of control over their children’s dietary and lifestyle environments such that they might be able to prevent health-risk behaviors and facilitate health-promoting behaviors. Therefore, researchers are more willing to engage parents on obesity management interventions.

In our study strengths and limitations of each psychological intervention designs and methods was presented. We think that the design-based research is an appropriate approach to creating feasible and more effective intervention programs that address overweight and obesity- as complex health problems. By understanding such details (strengths and limitations), researchers and professionals will be able to strengthen intervention implementation, evaluation, and sustainability. Besides, this part of our study will support researchers for identification of potential challenges as well. However, we think that there are differences between methodological ideals that we have presented here and the real-life intervention context. So, such differences should be determined in future studies. For example, proper implementation of CBT requires engaging trained employees and target groups who are willing to receive this type of therapy to achieve the maximum benefit for obesity management. Therefore, CBT may not generally be a cost-effective method for the health care system as well as the society in the long term. However, we know that weight loss is a complex problem that involves issues (e.g., body image, self-image, confidence, etc.,) so that CBT can be a useful psychological intervention in several stages of the weight management program. So, we argue that researchers or health authorities should make the final and case-specific decisions regarding implementation of this type of intervention based on their resources including human, financial resources, and time, etc. They should also think if the advantages outweigh the disadvantages for the target population as well as the healthcare system in the long run. MI as another psychological method respects the patient's autonomy and it will work properly for losing weight. Nevertheless, it requires trained staff in weight loss programs. So, we believe it might not be generally supported by the health sector especially primary health care providers. GPs with previous therapeutic training might be interested to apply such methods in weight management plans, though this should be tested in real-life interventions. Alternatively, staff specifically trained in these methods should be used.

In the present meta-analysis, we combined non-family interventions (e.g., children and teachers) with family-based interventions into a general group-based intervention format. The rationale for such a combination was the limited number of studies which had applied non-family intervention design. This might have affected the results of our study. Mainly because family dynamics (e.g., communication pattern, interaction between family members, etc.) as well as culture, family norms, parents’ beliefs and health perception might influence the effectiveness of weight management programs, while such influence might not be seen in non-family groups. For instance, parents who perceive their child’s overweight or obesity as a genetic inheritance might not feel the need to modify their unhealthy lifestyle [88], because they do not consider excess-weight or even obesity as a modifiable risk factor for overall health of their children. We believe that adverse effects of family characteristics on the efficacy of the interventions might affect even well-defined treatment plans. So, for the design and implementation of future theory-based treatment interventions, researchers, GPs, and other health authorities are encouraged to be more focused on family elements (e.g., family norms, parents’ health perception) associated with healthy behaviors. Eventually we argue that future interventions should be socially and culturally adapted for the target population to gain more success and sustainability in obesity management plan, however effectiveness of such approach should be trialed.

In our meta-analysis, we also evaluated effectiveness of 30 studies that included psychological interventions for obesity management. In a previous study, researchers could not make a firm conclusion about the effectiveness of psychological interventions for childhood obesity [10]. But our findings demonstrated benefits of psychological interventions specifically CBT and MI for obesity management. Therefore, our findings both fill the above-mentioned knowledge gap and specify the direction of future research which will be discussed in the following.

In terms of intervention components, our findings revealed that interventions with “moderate-intensity” are more effective than the other types of intervention intensity for reducing BMI and BMI z-score (generalized obesity). Meaning that, these kinds of interventions produced the largest reduction in BMI and BMI z-score among the studied population. However, WC (abdominal obesity) improved significantly with “low intensity” interventions. A previous study showed short-term benefits of comprehensive medium- to high-intensity behavioral interventions in children and adolescents with obesity [17]. The presently discussed interventions appear to offer effects that are durable for the follow-up period subsequent to the intervention. However, we argue that the combination of cognitive and motivational techniques with behavioral therapies with more focus on intervention components should be trialed in future studies because it will probably improve the maintenance of positive effects on obesity management.

### Strengths and limitations of the study

This is the first comprehensive study to investigate the effectiveness of multicomponent psychological interventions on managing overweight and obesity among school-aged children. However, the current study also has limitations. First, we excluded some of the studies from the meta-analysis due to a variety of reasons (e.g., lack of sufficient data on primary outcomes of obesity, not having a control group). So, due to sample size limitation we were unable to perform subgroup analyses for all obesity outcomes and this may affect our conclusion on the effect of the interventions. Second, the combination of non-family interventions with family-based interventions- as “group-based” interventions might also affect the results of our study as family and non-family-based groups might be unequal. Family groups are more likely to have a connection and some sort of shared identity than non-family groups have, which may dilute the overall effect of group-based interventions if these two group formats are combined and treated as one. Therefore, this important limitation should also be considered in future studies.

## Conclusion

Evaluated interventions showed benefits of CBT and MI as psychological interventions targeting school-aged children with overweight and obesity. Hence, such kind of interventions for weight management of our target population is recommended. It is also important that existing psychological interventions in conjunction with other treatment methods are tested and continually improved for more sustainability in obesity management as well as improvements on other health outcomes and quality of life. Furthermore, authors would like to suggest conducting more studies to explore and apply family characteristics, social elements or other factors affecting efficacy and adherence of the interventional programs. Finally, we suggest that differences between methodological ideals and the real-life intervention context should be determined in future studies by asking for feedback from GPs, professional healthcare providers, and patients who are engaged in the intervention implementation.

### Supplementary Information

**Additional file 1.** Search strategy.

**Additional file 2.** Characteristics of the included studies.

## Data Availability

The original contributions presented in the study are included in the article as supplementary material. Further inquiries can be directed to the corresponding authors.

## Abbreviations

NCDs: Non-Communicable diseases
CBT: Cognitive behavioral therapy
MI: Motivational interviewing
PRISMA-P: Preferred Reporting Items for Systematic Reviews and Meta-Analysis guideline
PROSPERO: International Prospective Register of Systematic Reviews
ISI/WOS: Web of Science
BMI: Body mass index
WC: Waist circumference
WhtR: Waist-to-height ratio
RCTs: Randomized controlled trials
QRCTs: Quasi-randomized controlled trials
RoB_2_: Cochrane Risk of Bias tool
QL: Quality of life
PA: Physical activity
SMD: Standardized mean differences
CI: Confidence interval
SE: Standard error
GPs: General practitioners
HC: Hip Circumferences
BP: Blood Pressure
LDL: Low Density Lipoprotein
HDL: High Density Lipoprotein
TG: Triglycerides
TC: Total Cholesterol
WHR: Waist to Hip Ratio
NP: Not Provided
