# Supplementary material for: The effect of psychological interventions targeting overweight and obesity in school-aged children: a systematic review and meta-analysis
Source: BMC Public Health. 2023 Aug 3;23:1478. doi: 10.1186/s12889-023-16339-7 (PMC10398924; doi:10.1186/s12889-023-16339-7)
Supplement: Supplementary file 1 — Additional file 1. Search strategy. [file 12889_2023_16339_MOESM1_ESM.docx]

**Additional file 1:** Search strategy

| **PubMed** |
| --- |
| (((("Obesity"[Mesh]) OR ("Overweight"[Mesh])) OR ((((obes*[Title/Abstract]) OR (overweight[Title/Abstract])) OR ("over nutrient"[Title/Abstract])) OR (Overeating[Title/Abstract]))) AND ((("Child"[Mesh]) OR ("Schools"[Mesh])) OR (((((((Child[Title/Abstract]) OR (preschool[Title/Abstract])) OR (young[Title/Abstract])) OR (youth[Title/Abstract])) OR (teen[Title/Abstract])) OR (adolescent[Title/Abstract])) OR (pediatric[Title/Abstract])))) AND (((("Psychosocial Intervention"[Mesh]) OR ("Behavior Therapy"[Mesh])) OR ("Cognitive Behavioral Therapy"[Mesh])) OR (((((("Behavior Therapy"[Title/Abstract]) OR ("Behavior Treatment"[Title/Abstract])) OR ("Conditioning Therapy"[Title/Abstract])) OR ("Behavior Modification"[Title/Abstract])) OR ("multi-component program"[Title/Abstract])) OR ("parenteral intervention"[Title/Abstract]))) |
| **Scopus** |
| ( ( TITLE-ABS-KEY ( "Psychosocial Intervention" )  OR  TITLE-ABS-KEY ( "Behavior Therapy" )  OR  TITLE-ABS-KEY ( "Cognitive Behavioral Therapy" )  OR  TITLE-ABS-KEY ( "Behavior Therapy" )  OR  TITLE-ABS-KEY ( "Behavior Treatment" )  OR  TITLE-ABS-KEY ( "Conditioning Therapy" )  OR  TITLE-ABS-KEY ( pediatric )  OR  TITLE-ABS-KEY ( "Behavior Modification" )  OR  TITLE-ABS-KEY ( "multi-component program" )  OR  TITLE-ABS-KEY ( "parenteral intervention" ) ) )  AND  ( ( ( TITLE-ABS-KEY ( obes* )  OR  TITLE-ABS-KEY ( overweight )  OR  TITLE-ABS-KEY ( "over nutrient" )  OR  TITLE-ABS-KEY ( overeating ) ) )  AND  ( ( TITLE-ABS-KEY ( child* )  OR  TITLE-ABS-KEY ( school )  OR  TITLE-ABS-KEY ( young )  OR  TITLE-ABS-KEY ( youth )  OR  TITLE-ABS-KEY ( teen* )  OR  TITLE-ABS-KEY ( adolescent )  OR  TITLE-ABS-KEY ( pediatric ) ) ) )  AND  ( LIMIT-TO ( SUBJAREA ,  "PSYC" )  OR  LIMIT-TO ( SUBJAREA ,  "SOCI" )  OR  LIMIT-TO ( SUBJAREA ,  "MULT" ) ) |
| **ISI/WOS** |
| TOPIC: (Obes*) OR TOPIC: (Overweight) OR TOPIC: ("over nutrient") OR TOPIC: (Overeating)  Indexes=SCI-EXPANDED, SSCI, A&HCI, CPCI-S, CPCI-SSH, BKCI-S, BKCI-SSH, ESCI Timespan=All years  TOPIC: (child*) OR TOPIC: (school) OR TOPIC: (preschool) OR TOPIC: (young) OR TOPIC: (youth) OR TOPIC: (teen8) OR TOPIC: (adolescent [) OR TOPIC: (pediatric)  Indexes=SCI-EXPANDED, SSCI, A&HCI, CPCI-S, CPCI-SSH, BKCI-S, BKCI-SSH, ESCI Timespan=All years  TOPIC: ("Psychosocial Intervention") OR TOPIC: ("Behavior Therapy") OR TOPIC: ("Cognitive Behavioral Therapy") OR TOPIC: ("Behavior Therapy") OR TOPIC: ("Behavior Treatment") OR TOPIC: ("Conditioning Therapy") OR TOPIC: ("Behavior Modification") OR TOPIC: ("multi-component program") OR TOPIC: ("parenteral intervention")  Indexes=SCI-EXPANDED, SSCI, A&HCI, CPCI-S, CPCI-SSH, BKCI-S, BKCI-SSH, ESCI Timespan=All years  #3 AND #2 AND #1  Indexes=SCI-EXPANDED, SSCI, A&HCI, CPCI-S, CPCI-SSH, BKCI-S, BKCI-SSH, ESCI Timespan=All years |
