# Supplementary material for: The effect of psychological interventions targeting overweight and obesity in school-aged children: a systematic review and meta-analysis
Source: BMC Public Health. 2023 Aug 3;23:1478. doi: 10.1186/s12889-023-16339-7 (PMC10398924; doi:10.1186/s12889-023-16339-7)
Supplement: Supplementary file 2 — Additional file 2. Characteristics of the included studies. [file 12889_2023_16339_MOESM2_ESM.docx]

**Additional file 2:** Characteristics of the included studies

| **Study** | **Sample Size** | **Age (Year), Mean/Range** | **Psychological Intervention/Theory** | **Intervention Duration (Months)** | **Outcomes** | **Follow-up time (Months)** | **Risk of Bias** |
| --- | --- | --- | --- | --- | --- | --- | --- |
| Janicke et al, (2019), USA ^[18]^ | 249 | 8-12 | CBT | 12 | BMI z-score, Weight | 24 | Low |
| Miri et al, (2019), Iran ^[19]^ | 110 | 13-18 | CBT | 6 | Height, Weight, WC, HC, Body fat | 6 | High |
| Moore et al, (2019), USA ^[20]^ | 360 | 11.6 | CBT& MI | 36 | BMI, WC, BP, LDL, HDL, TG, TC | *Multiple | Low |
| Sepúlveda et al (2019), Spain ^[21)^ | 70 | 8-12 | CBT& MI | 6 | BMI z-score | 6 | Some concerns |
| Wilson et al (2019), USA ^[22]^ | 549 | 2-12 | CBT | 12 | Parent feeding practices | 12 | Some concerns |
| Bean et al, (2018), USA ^[23]^ | 99 | 11-18 | MI | 6 | Height, weight, BMI z score | Multiple | Some concerns |
| Crespo et al, (2018), USA ^[24]^ | 297 | 5-10 | Socioecological Model& MI | 12 | BMI | Multiple | Low |
| Njardvik et al, (2018), Iceland ^[25]^ | 84 | 8-12 | MI | 4.5 | Weight, BMI-SDS | NP | Some concerns |
| Sen et al (2018), Turkey ^[26]^ | 108 | 9-12 | MI | 3 | BMI& BMI z-score | NP | Some concern |
| Yackobovitch-Gavan et al (2018), Israel ^[27]^ | 270 | 5-11 | CBT | 3 | BMI, BP, TG, HDL, TC | 24 | High |
| Annesi et al, (2017), USA, ^[28]^ | 141 | 9-12 | CBT | 6 | Height, weight, BMI z score | 9 | Low |
| Bagherniya et al, (2017), Iran ^[29]^ | 172 | 12-16 | Social cognitive theory | 7 | BMI, WC | 7 | Some concerns |
| Wilfley et al (2017), USA ^[30]^ | 172 | 7-11 | CBT& Socioecological approach | 8 | Weight, Height, BMI percentile | 12 | Some concerns |
| Larsen et al, (2016), Denmark ^[31]^ | 106 | 11-13 | Psychosocial model of planned behavior change & Trans-theoretical model at the individual level | 1.5 | BMI z-score, Body  composition | 4.5 | High |
| Daly et al, (2016), USA ^[32]^ | 37 | 14-17 | Information-Motivation-Behavioral Skills Theory | 1.5 | Height, Weight, BMI | 2.5 | Low |
| Kulendran et al, (2016), UK ^[33]^ | 27 | 13.7 | NP | 3 | BMI | NP | Some concerns |
| Pbert et al, (2016), USA ^[34]^ | 126 | 16.5 | CBT | 8 | BMI | 8 | Low |
| Larsen et al, (2015), Denmark ^[35]^ | 80 | 5-9 | Small steps & realistic goals approach | 24 | BMI z score | 24 | Low |
| Kulik et al, (2015), USA ^[36]^ | 41 | 13-17 | CBT | 4 | Weight, Height, BMI | NP | Low |
| Norman et al, (2015), USA ^[37]^ | 106 | 11-13 | Chronic Care Model& Social cognitive theory | 12 | BMI | Multiple | Some concerns |
| Parra-Medina et al, (2015), USA ^[38]^ | 118 | 5-14 | NP | 4.5 | Weight, WC, BMI | Multiple | Low |
| Serra-Paya et al (2015), China ^[39]^ | 113 | 6-12 | NP | 8 | Anthropometric measurements | NP | Low |
| Taveras et al (2015), USA ^[40]^ | 549 | 6-12 | MI | 9 | BMI | 12 | Low |
| Steele et al (2014), USA ^[41]^ | 93 | 7-17 | NP | 2.5 | Height, Weight, BMI z-score | NP | High |
| van der Baan-Slootweg et al (2014), Netherlands ^[42]^ | 90 | 8-18 | CBT | 6 | BMI z-score, WC, BP, body composition | 12 | Low |
| Wright et al (2014), USA ^[43]^ | 251 | 8-12 | Community–academic partnered participatory research (CPPR) | 4 | Weight, Height, BMI, BP, WC | Multiple | Some concerns |
| Danielsen et al, (2013), Norway ^[44]^ | 49 | 7-13 | CBT | 3 | BMI, BMI SDS | 12 | High |
| Davis et al, (2013), USA ^[45]^ | 58 | 5-11 | MI | 8 | Height, weight, BMI z score | NP | Some concerns |
| Johnston et al, (2013), USA ^[46]^ | 71 | 10-14 | Behavioral strategies | 6 | BMI, BMI percentile | Multiple | Some concerns |
| Lochrie et al, (2013), USA ^[47]^ | 130 | 8-11 | NP | 6 | BMI z-score, WC, BP | 12 | Low |
| O’Connor et al, (2013), USA ^[48]^ | 40 | 5-8 | Social cognitive& Parenting theories | 6 | BMI | 6 | Some concerns |
| Saelens et al (2013), USA ^[49]^ | 72 | 7-11 | MI& Autonomy enhancing | 5 | Anthropometric measurements | Multiple | Low |
| Verbeken et al (2013), Belgium ^[50]^ | 44 | 8-14 | CBT | 1.5 | BMI | 3 | Low |
| Wright et al (2013), USA ^[51]^ | 50 | 9-12 | NP | 3 | BMI percentile, BMI z-score | 3 | Low |
| Nemet et al, (2013), Israel ^[52]^ | 40 | 8.5 ± 1.2 | NP | 3 | Weight, BMI, BMI percentile | NP | Low |
| Croker et al, (2012), UK ^[53]^ | 72 | 8-12 | Learning Theory& MI | 6 | Waist, BMI, BP | 12 | Some concerns |
| Fullerton et al, (2012), USA ^[54]^ | 80 | 12.1 | NP | 6 | BMI | NP | High |
| Lloyd et al, (2012), USA ^[55]^ | 118 | 13-16 | CBT | 4.5 | BMI | Multiple | Some concerns |
| Nguyen et al, (2012), Australia ^[56]^ | 151 | 13-16 | CBT | 12 | BMI z-score, WHR | 12 | Low |
| Toulabi et al (2012), Iran ^[57]^ | 152 | 15.7 | NP | 6 | WC, HC, WrC, BMI, WHR | NP | Some concerns |
| Vos et al (2012), Netherlands ^[58]^ | 81 | 8-17 | CBT | 3 | BMI | 12 | Some concerns |
| Díaz et al, (2010), Mexico ^[59]^ | 76 | 9-17 | Health belief Model | 12 | BMI | Multiple |  |
| Reinehr et al, (2010), Germany ^[7]^ | 66 | 8-16 | NP | 6 | BMI, BP, WC | 6 | High |
| Kalarchian et al, (2009), USA ^[60]^ | 192 | 8-12 | MI | 6 | BP, Body composition, WC | Multiple | Some concerns |
| Kitzman-ulrich et al, (2009), USA ^[61]^ | 42 | 12-15 | CBT& Family Systems theories | 4 | BMI | 4 | Some concerns |
| Sarvestani et al (2009), Iran ^[62]^ | 60 | 11-15 | Behavioral Strategies | 6 | BMI | NP | Some concerns |
| Hughes et al, (2008), UK ^[63]^ | 134 | 5-11 | Behavioral change models& MI | 6.5 | BMI z score, weight | Multiple | Low |
| Munsch et al, (2008), Switzerland ^[64]^ | 56 | 8-12 | CBT | 6 | BMI | 6 | Low |
| Savoye et al (2007), USA ^[65]^ | 209 | 8-16 | CBT | 12 | BMI, body fat | Multiple | High |
| Williamson et al (2006), USA ^[66]^ | 57 | 13.2 | MI | 24 | BMI, Body composition | Multiple | Low |
| Jiang et al, (2005), China ^[67]^ | 68 | 13.2 | MI | 24 | BP, TC, TG | Multiple | Low |
| Herrera et al, (2004), USA ^[68]^ | 75 | 6-18 | MI& CBT | 2.5 | BMI | Multiple | Some concerns |
| Goldfield et al, (2001), USA ^[69]^ | 31 | 8-12 | MI | 6 | BMI z-score | Multiple | Some concerns |
| Epstein et al, (1994), USA ^[70]^ | 44 | 8-12 | MI | 6 | BMI | Multiple | Some concerns |
| ***Multiple:** More than one follow-up data was provided.  **CBT:** Cognitive Behavior Therapy, **BMI:** Body Mass Index, **MI:** Motivational Interviewing, **WC:** Waist Circumference, **HC:** Hip Circumferences, **BP:** Blood Pressure, **LDL:** Low  Density Lipoprotein, **HDL:** High Density Lipoprotein, **TG:** Triglycerides, **TC:** Total Cholesterol, **WrC:** Wrist Circumferences, **WHR:** Waist to Hip Ratio, **NP:** Not Provided | | | | | | | |
